# Microbiota dysbiosis and its pathophysiological significance in bowel obstruction

**DOI:** 10.1038/s41598-018-31033-0

**Published:** 2018-09-03

**Authors:** Shrilakshmi Hegde, You-Min Lin, George Golovko, Kamil Khanipov, Yingzi Cong, Tor Savidge, Yuriy Fofanov, Xuan-Zheng Shi

**Affiliations:** 1 Department of Internal Medicine, University of Texas Medical Branch, Galveston, TX USA; 2 Department of Pharmacology & Toxicology, University of Texas Medical Branch, Galveston, TX USA; 3 Department of Microbiology & Immunology, University of Texas Medical Branch, Galveston, TX USA; 4 Department of Pathology & Immunology, Baylor College of Medicine, Houston, TX USA

## Abstract

Bowel obstruction (OB) causes local and systemic dysfunctions. Here we investigated whether obstruction leads to alterations in microbiota community composition and total abundance, and if so whether these changes contribute to dysfunctions in OB. Partial colon obstruction was maintained in rats for 7 days. The mid colon and its intraluminal feces - proximal to the obstruction - were studied. OB did not cause bacterial overgrowth or mucosa inflammation, but induced profound changes in fecal microbiota composition and diversity. At the phylum level, the 16S rRNA sequencing showed a significant decrease in the relative abundance of *Firmicutes* with corresponding increases in *Proteobacteria* and *Bacteroidetes* in OB compared with sham controls. Daily treatment using broad spectrum antibiotics dramatically reduced total bacterial abundance, but increased the relative presence of *Proteobacteria*. Antibiotics eliminated viable bacteria in the spleen and liver, but not in the mesentery lymph node in OB. Although antibiotic treatment decreased muscle contractility in sham rats, it had little effect on OB-associated suppression of muscle contractility or inflammatory changes in the muscle layer. In conclusion, obstruction leads to marked dysbiosis in the colon. Antibiotic eradication of microbiota had limited effects on obstruction-associated changes in inflammation, motility, or bacterial translocation.

## Introduction

Obstructive bowel disorders (OBD) are characterized by intraluminal retention and lumen distention due to mechanical or functional obstruction (OB) in the gut^1,2^. Mechanical OB is caused by numerous reasons, and may originate outside the intestine (e.g. adhesions and hernias), or inside (e.g., carcinoma and diverticulitis). Adhesions and hernias are the most common causes of small bowel obstruction, whereas carcinoma and diverticulitis constitute most of the causes for colon obstruction^3–5^. Functional OB results from neuromuscular dysfunction, such as in ileus, intestinal pseudo-obstruction, idiopathic megacolon, and Hirschsprung’s disease^3,5–7^. OBD represent a significant health challenge in adults and children^2–5^. Mechanical OB, as the most common type of OBD^4,5,8^, accounts for more than 300,000 hospital admissions per year in the US alone^8^. The aggregate annual cost of hospitalization for mechanical OB ($2.7 billion) exceeds all other gastrointestinal (GI) conditions, including hemorrhage, appendicitis and ulcers^8^.

Bowel obstruction causes a series of local and systemic changes^3,5^. Gut motility dysfunction is one of the most prominent local pathological changes in OB and is responsible for symptoms such as abdominal distention, nausea, vomiting, and constipation^3,5^. Moderate gut inflammation is reported in OB^3,9,10^, although what causes inflammatory changes is not very clear. OB may also lead to severe systemic responses, such as sepsis and septic shock^3,5^. Increased bacterial translocation in OB is considered a major pathogenic factor for the systemic responses^3,5^. However, the mechanisms underlying the local and systemic alterations in OB are still not well understood.

Gut microbiota is considered a second genome that actively modulates human health^11^. With over 100 trillion microbial cells, the gut microbiota influences host’s GI physiology, metabolism, nutrition and immune function^12,13^. Changes in microbiota composition, quantity, diversity, and metabolic activity (dysbiosis) have been described in GI conditions such as inflammatory bowel disease (IBD), irritable bowel syndrome (IBS), obesity, and colorectal cancer^14–19^. It is believed that dysbiosis, i.e. changes in microbiota composition may reflect a compensatory response to maintain human health, whereas specific alterations in microbiota quantity, composition and diversity may have different pathological consequences^20,21^.

Bowel obstruction with increased fecal retention has long been thought to promote microbiome overgrowth, i.e. bacterial proliferation^22^. This is largely based on studies using bacteria culture. Furthermore, bacterial proliferation is considered to contribute to local^22,9^ and systemic responses in OB^23^. Hence, treatment of obstruction often includes broad-spectrum antibiotics^23^. However, how OB affects microbiota community dynamics and whether the microbiota alterations contribute to pathophysiological changes are largely unknown.

Culture-independent techniques developed over the last decade have made it possible for more accurate and sophisticated assessment of gut microbiota community dynamics^24–26^ and have generated new opportunities to better understand GI disease^27^. In the present study, we systemically investigated microbiota quantity, composition and diversity in mechanical OB using multiple approaches, i.e. culture-independent 16S rRNA sequencing and 16S rRNA qPCR, as well as culture-dependent viable counts. We also investigated the effect of broad-spectrum antibiotics on microbiota composition in OB. Finally, we evaluated the effect of antibiotics as a treatment choice for microbiota elimination on colon smooth muscle contractility, inflammatory changes, and bacterial translocation in OB to determine whether the microbiota plays a role in these dysfunctions.

## Results

### Effect of obstruction on total bacterial abundance in the colon

**Figure 1:**
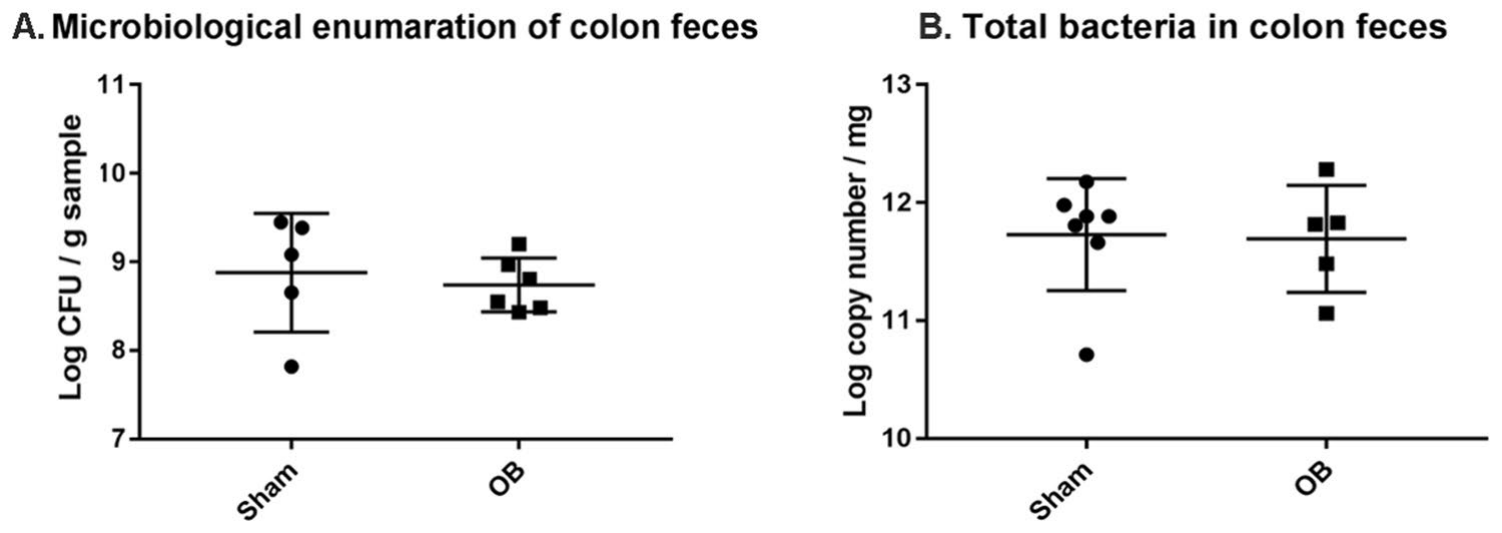
Quantification of total bacteria abundance in colon feces of sham and obstruction (OB) rats. Total viable bacteria (anaerobic and aerobic) in fresh colon feces were quantified by plating serial dilutions on GAM agar (for anaerobic bacteria) and Tryptic soy agar (for aerobic). The total CFU of bacteria/gram sample is shown in (**A**) (Sham, n = 5; OB, n = 6). The 16S rRNA copy numbers in colon feces was calculated by RT-PCR and data is presented as total 16S rRNA copy numbers/mg sample ± standard deviation (**B**) (sham, n = 7; OB, n = 5). *p* > 0.05 vs. sham (unpaired *t*-test).

We first determined the total bacterial load in the colonic feces of sham and OB rats. Microbiological analysis revealed no significant difference in total viable bacteria (anaerobes and aerobes) per unit wet weight between sham [(1.13 ± 1.21) × 10^9^ CFU g^−1^, n = 5] and OB rats [(7.73 ± 6.23) × 10^8^ CFU g^−1^, n = 6, *p* > 0.05] (Fig. 1A). The 16 s rRNA qPCR analysis also found no significant difference in the total 16S rRNA gene copy number between OB [log (11.6 ± 0.45) copies mg^−1^, n = 7] and sham [log (11.7 ± 0.47) copies mg^−1^, n = 5, *p* > 0.05] (Fig. 1B). Although colon contents in OB rats were 2.66-fold larger than those in sham controls, the total 16S rRNA copies in OB [Log (15.6 ± 0.45)] was not significantly greater than that in sham [Log (15.2 ± 0.47), *p* > 0.05].

### Microbiota composition and diversity in obstruction

**Figure 2:**
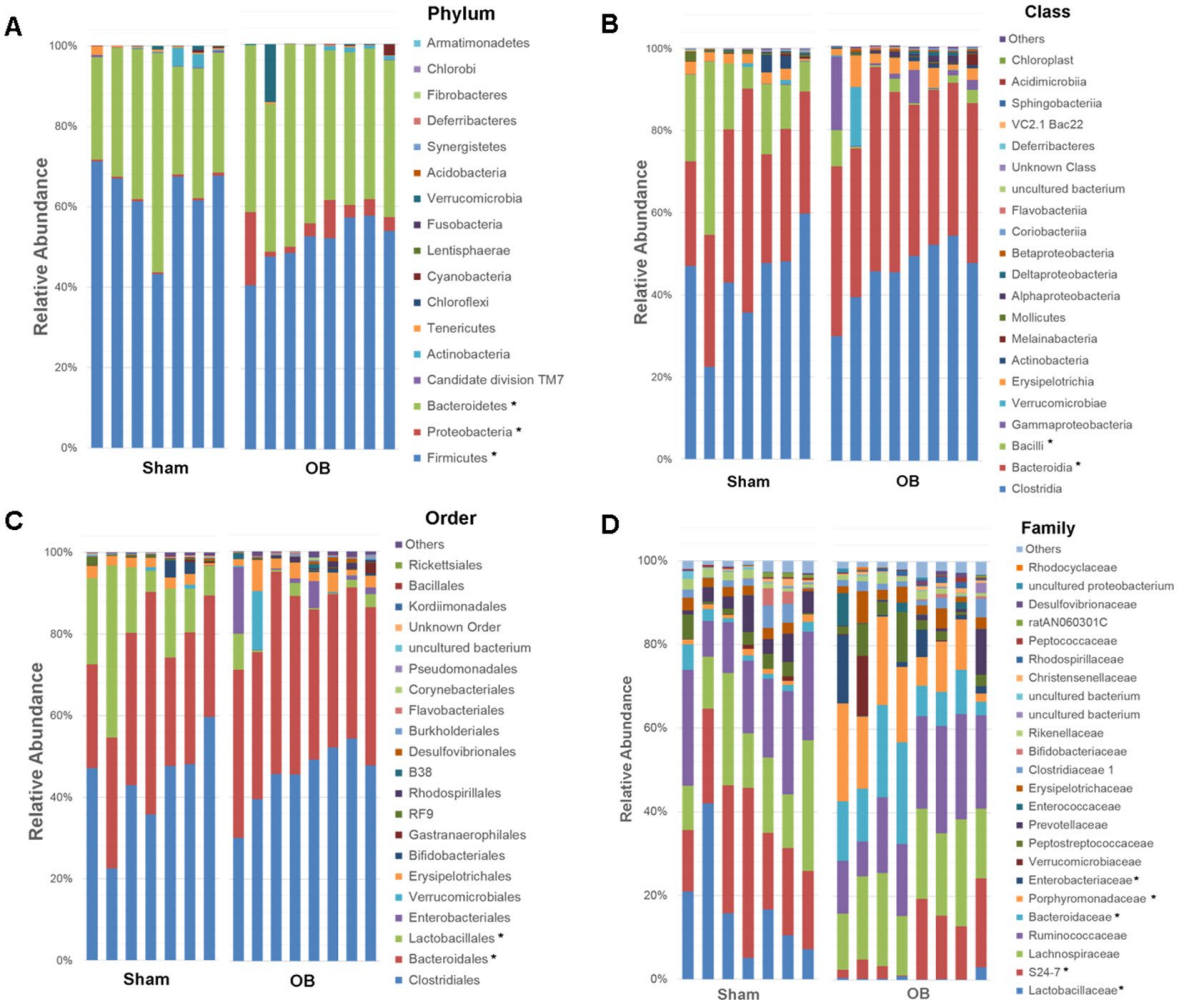
Comparison of Colon microbiota composition in sham and OB rats: Total DNA was extracted from colon feces collected from sham (n = 7) and OB rats (n = 8), and 16S rRNA high throughput sequencing was carried out as described in Methods. Colon microbiota composition at Phylum level (**A**), Class level (**B**), Order level (**C**) and Family level (**B**) are shown in the figure. *Indicates the taxa with significantly different abundance between sham and OB (2-way ANOVA, *p* < 0.05).

We then used 16S rRNA sequencing to assess bacterial microbiota composition in the colon feces of 7 sham and 8 OB rats, and found that obstruction led to microbiota community shifts at all taxonomical levels including phylum, class, order, family, and genus (Fig. 2A–D). At the phylum level, there was a significant decrease in the relative abundance of *Firmicutes* in OB compared to sham [Sham: 62.8% (±9.3), OB: 51.2% (±5.6), *p* < 0.0001]. However, OB caused significant increases in the relative abundance of *Bacteroidetes* [Sham: 34.0% (±4.6), OB: 40.2% (±9.8), *p* = 0.001] (Fig. 2A) and *Proteobacteria* [Sham: 0.49% (±0.13), OB: 5.5% (±5.6), *p* = 0.01] (Fig. 2A). At the class level, the abundance of *Bacilli* decreased in OB (*p* < 0.0001), whereas *Bacteroidia* increased significantly (*p* = 0.007) (Fig. 2B). We also observed relative increases in *α-proteobacteria*, *β-proteobacteria*, *γ-proteobacteria* and *Flavobacteria* in OB (Fig. 2B). At the order level, the abundance of *Lactobacillales* decreased and *Bacteroidales* increased in OB (*p* < 0.05) (Fig. 2C). At the family level, differential abundance was observed in *Lactobacillaceae*, *S24-7, Bacteroidaceae*, *Porphyromonadaceae*, and *Enterobacteriaceae* (*p* < 0.05) (Fig. 2D). At the genus level, we identified 9 genera with differential abundance in OB (*p* < 0.05) (data is summarized in Fig. S1).

**Figure 3:**
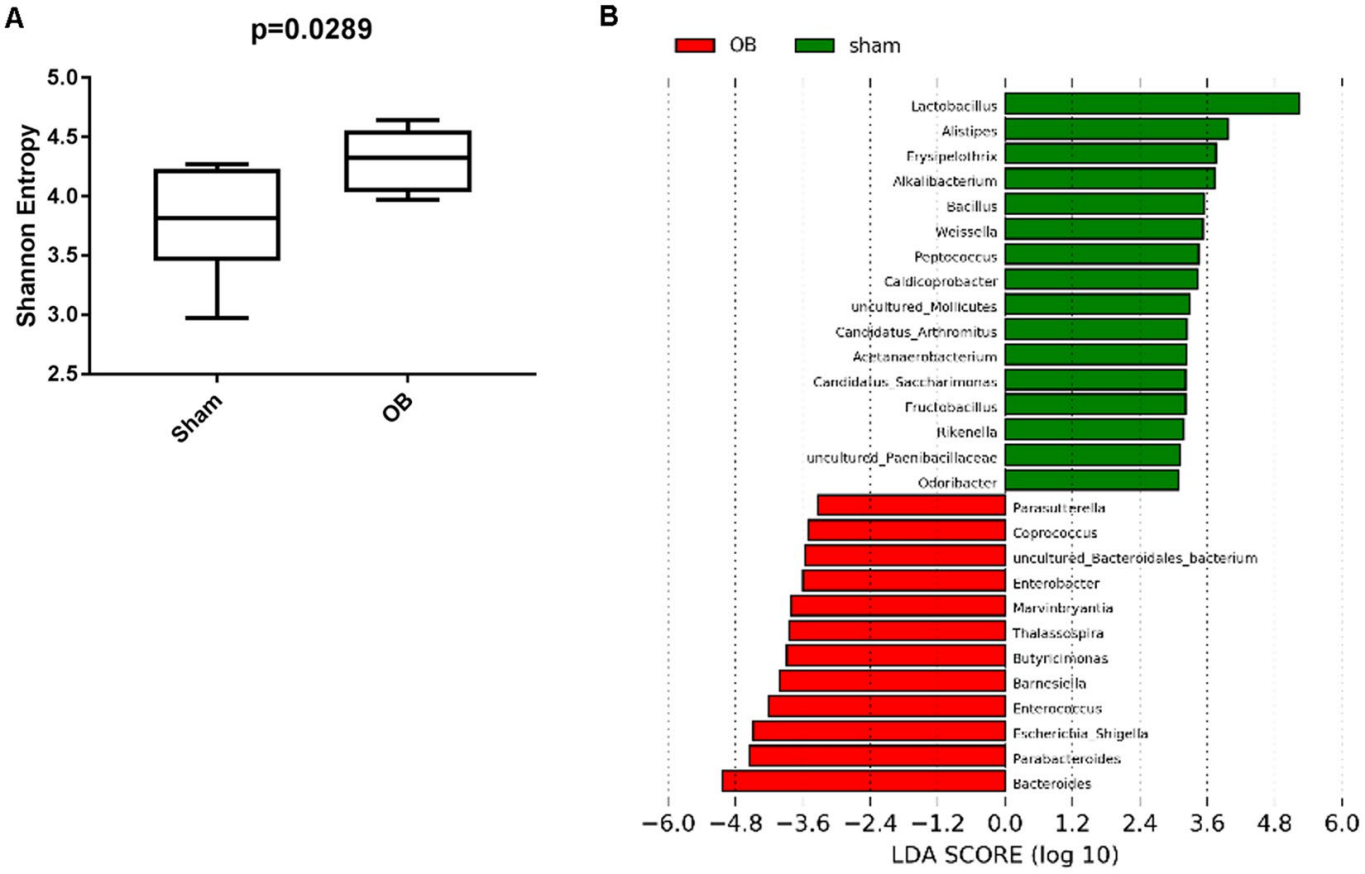
Alpha diversity and LEfSe analysis of fecal contents from sham and obstruction rats at genera level. Alpha diversity at genera level is represented in terms of Shannon diversity index and statistical significance was calculated using Mann-Whitney test and *p* < 0.05 is considered significant (**A**). LEfSe analysis for effect size measurement of differentially abundant genera in OB compared to sham rats is depicted in (**B**). Genera enriched in sham are represented in positive LDA score (green) and genera enriched in obstruction condition are represented in negative LDA score (red).

We also calculated microbiota alpha diversity at the genera level between sham and OB samples, using Shannon Diversity Index (SDI) (Fig. 3A). There is a significantly higher SDI in OB samples than in sham (*p* = 0.02), indicating a greater microbiota diversity in obstructed colon (Fig. 3A).

To further identify the differentially abundant and biologically relevant genera in OB, we carried out a LEfSe analysis. LEfSe calculates the effect size of each differentially abundant genus through metagenomics biomarker discovery analysis^28^. Using LEfSe, we identified 28 genera whose abundance varied significantly between sham and OB (*p* < 0.05, LDA score > 2) (Fig. 3B). Microbiota in OB had higher levels of *Bacteroides, Parabacteroides, Barnesiella, Butrycimonas* (belonging to *Bacteroidetes* phylum) and *Escherichia-Shigella, Thalassospira, Enterobacter, Parasutterella* (belonging to *Proteobacteria*). In sham control samples, there were 16 different genera which had higher abundance. A majority of these genera belong to the *Firmicute* phylum (Fig. 3B).

### Effects of antibiotic treatment on microbiota abundance, composition, and diversity in sham and obstruction

**Figure 4:**
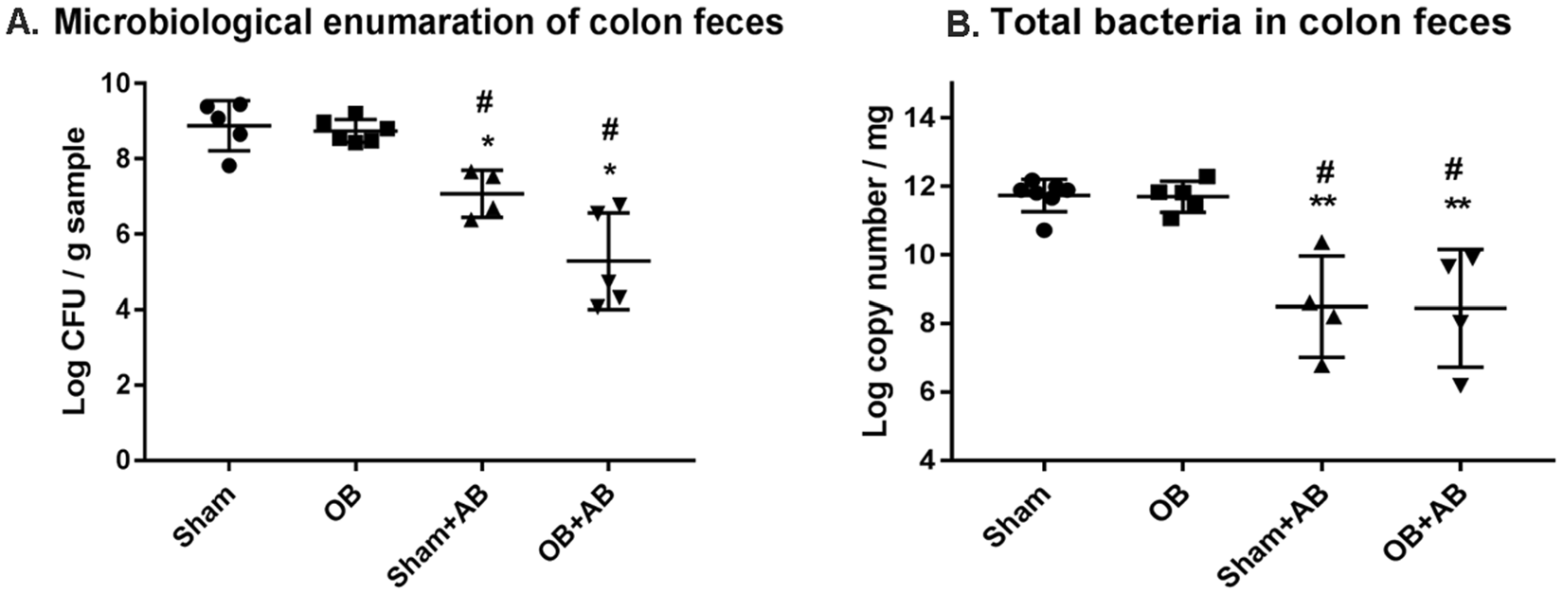
Quantification of total bacterial abundance in the colon feces of sham and OB rats after antibiotic treatment: Total viable bacteria (anaerobic and aerobic) were quantified from fresh colon feces taken from sham and OB rats without (sham and OB) and with antibiotic treatment (sham + AB and OB + AB). Total CFU of bacteria/gram sample taken ± standard deviation is shown here (**A**) (Sham, n = 5; OB, n = 6; sham + AB, n = 4; OB + AB, n = 5). Total 16S rRNA copy numbers were calculated using RT-PCR from colon feces collected from sham and OB rats treated without or with antibiotics (**B**) (Sham, n = 7; OB, n = 5; sham + AB, n = 4; OB + AB, n = 5). Data is presented as total 16S rRNA copy numbers/mg sample ± standard deviation. *p < 0.01 Vs sham, **p < 0.001 Vs sham, ^#^*p* < 0.003 Vs OB (1-way ANOVA).

To assess the possible role of microbiota in bowel function and the systemic response, we orally treated sham and OB rats with a broad spectrum antibiotic cocktail (+AB) to eliminate gut luminal bacteria. The antibiotic treatment started 1 day before operation, and continued for another 7 days until rats were euthanized. Sham and OB animals without antibiotic treatment were used as respective controls. Viable counts of total bacteria revealed that antibiotic treatment dramatically lowered the bacterial abundance in both sham [sham control: (1.13 ± 1.21) × 10^9^ CFU g^−1^; sham + AB: (1.78 ± 2.45) × 10^7^ CFU g^−1^, *p* < 0.02] and OB rats [OB control: (7.73 ± 6.23) × 10^8^ CFU g^−1^; OB + AB: (2.37 ± 2.86) × 10^6^ CFU g^−1^; *p* = 0.001] (Fig. 4A). The qPCR analysis found that antibiotic treatment resulted in a dramatic reduction of total 16S rRNA copies in both sham [sham control: log (11.7 ± 0.47) copies mg^−1^, sham + AB: log (8.48 ± 1.4) copies mg^−1^, *p* < 0.001] and OB rats [OB control: log (11.6 ± 0.45) copies mg^−1^, OB + AB: log (8.44 ± 1.7) copies mg^−1^, *p* < 0.001)] (Fig. 4B).

**Figure 5:**
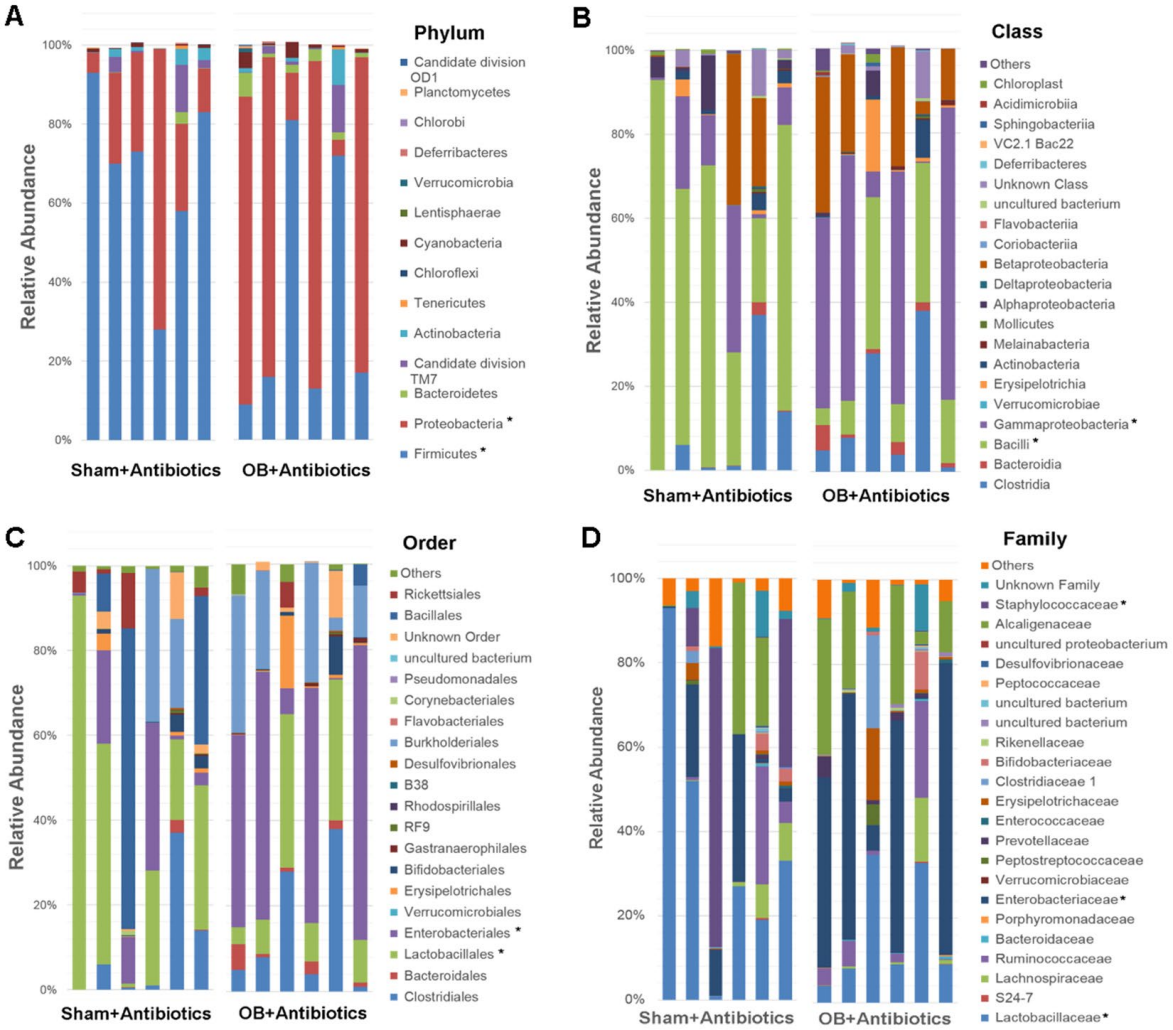
Comparison of Colon microbiota composition in sham and OB rats after antibiotic treatment. Total DNA was extracted from colon feces collected from sham and OB rats after antibiotic treatment (Sham + AB and OB + AB) and 16S rRNA high throughput sequencing was carried out. The graphs show the colon microbiota composition at Phylum level (**A**), Class level (**B**), Order level (**C**) and Family level (**D**). (sham + AB, n = 6; OB + AB, n = 6). *Indicates the taxa with significantly different abundance between the two groups (2-way ANOVA, *p* < 0.05).

We further compared microbiota community composition in control and antibiotics treated animals at different phylogenetic levels (Figs 2A–D and 5A–D). At the phylum level, antibiotic treatment increased the relative abundance of *Proteobacteria* in both sham and OB rats compared to the controls [sham: 0.49% (±0.13), sham + AB: 26.1% (±23.3); OB: 5.5% (±5.6), OB + AB: 56.3% (±37.5), (*p* < 0.0001)]. In contrast, the abundance of *Bacteroidetes* decreased significantly after antibiotic treatment in both sham [sham: 34.0% (±9.8), sham + AB: 0.57% (±1.19), *p* < 0.0001] and OB [OB: 40.2% (±4.6), OB + AB: 2.4% (±1.9), *p* < 0.0001]. The abundance of *Firmicutes* was not significantly affected by antibiotics in sham, but decreased in OB (*p* = 0.02). Antibiotic treatment also significantly altered the abundance of *Clostridia*, *Bacteroidia and γ-proteobacteria* class of bacteria in sham and OB compared to respective untreated counterparts (*p* < 0.05).

Compared to antibiotics treated sham controls, the antibiotics treated OB animals demonstrated significantly increased abundance of *Proteobacteria* and decreased *Firmicutes* at phylum level (*p* < 0.0001) (Fig. 5A), increased *γ-proteobacteria* and decreased *Bacilli* at class level (*p* < 0.0001) (Fig. 5B), increased *Enterobacteriales and* decreased *Lactobacillales* at order level (*p* < 0.0001) (Fig. 5C), and increased abundance of *Enterobacteriaceae* and *Staphylococcaceae* and decreased *Lactobacillaceae* at family level (*p* < 0.0001) (Fig. 5D). These results suggest that the dysbiosis observed in OB persisted even after the antibiotic treatment.

Antibiotic treatment significantly reduced bacterial diversity (represented by SDI) in both sham and OB rats (Fig. S2A,B) (*p* < 0.01). Among the antibiotics treated animals, there was no significant difference in the SDI values between sham and OB rats (Fig. S2C).

### Effect of antibiotic elimination of microbiota on bacterial translocation in OB

**Figure 6:**
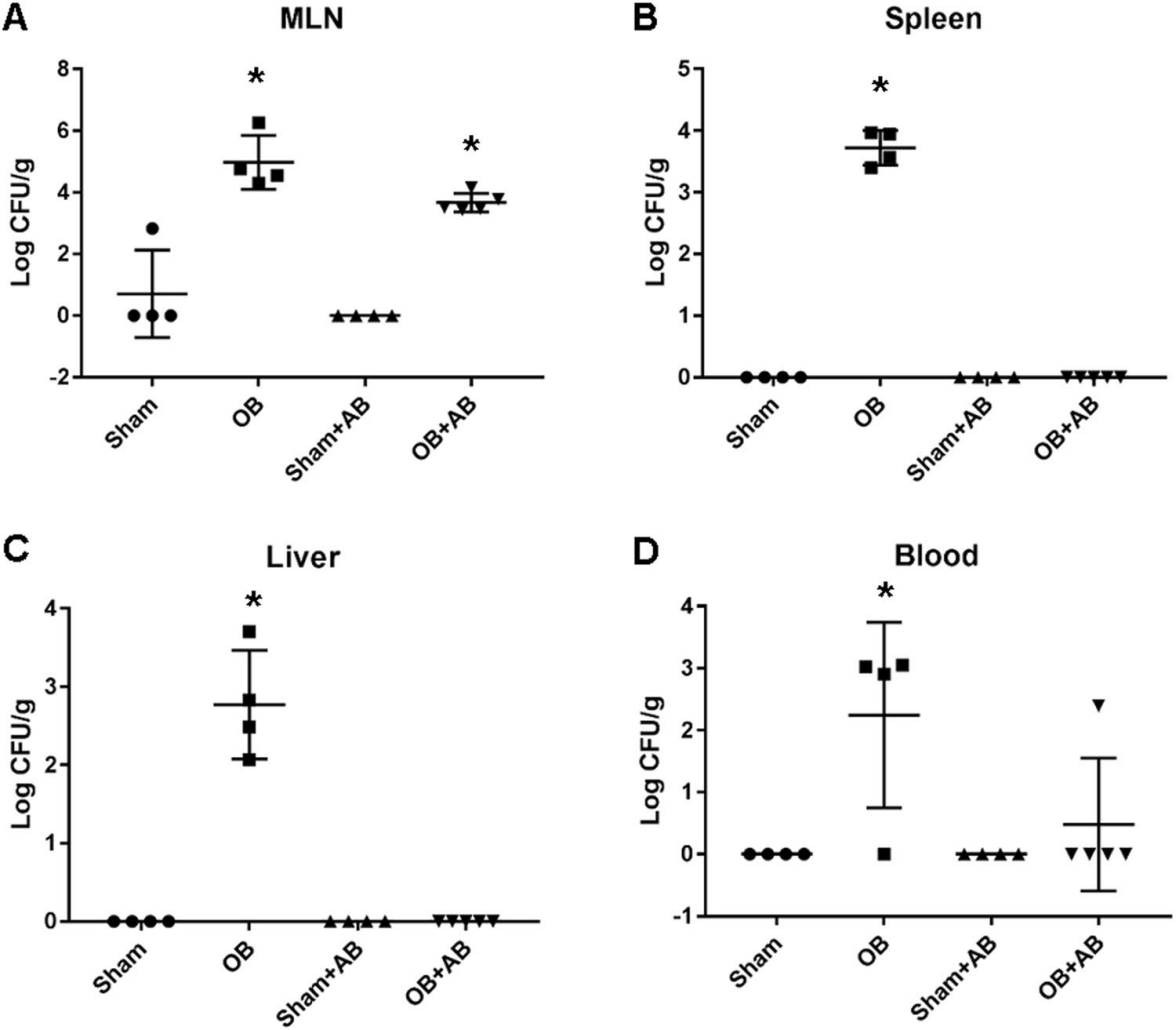
Bacterial translocation in sham and OB rats without and with antibiotic treatment. Bacterial translocation into the mesenteric lymph nodes (MLN) (**A**), spleen (**B**), liver (**C**) and blood (**D**) was assessed using tissue culture method. Total CFU of bacteria/gram sample (mean ± standard deviation) is shown here. *p < 0.05 Vs sham (1-way ANOVA), n = 4 except for OB + AB where n = 5.

Bacterial translocation from the gut to the mesenteric lymph node (MLN), visceral organs and blood was investigated by culture of viable organisms from tissues. We observed positive bacterial translocation to the MLN, spleen, liver and blood in the OB samples (N = 4). However, all the samples from sham controls (N = 4) remained negative except one sham rat in whom low abundance of bacteria was found in the MLN (Fig. 6A–D). The MLN and spleen had highest bacterial abundance in OB with (4.7 ± 8.7) × 10^5^ CFU gram^−1^ sample and (6.0 ± 3.4) × 10^3^ CFU gram^−1^ sample, respectively (*p* < 0.05 vs. sham).

After antibiotic treatment, bacterial counts in the spleen and liver in OB were eliminated (Fig. 6B,C). However, positive culture was still present in the blood in 1 of the 5 OB rats. In contrast, antibiotics failed to eliminate bacterial translocation to the MLN, from which we recovered (5.8 ± 4.8) × 10^3^ CFU of bacteria g^−1^ sample (Fig. 6A).

### Effects of antibiotics on inflammatory changes in the colon in sham and OB rats

**Figure 7:**
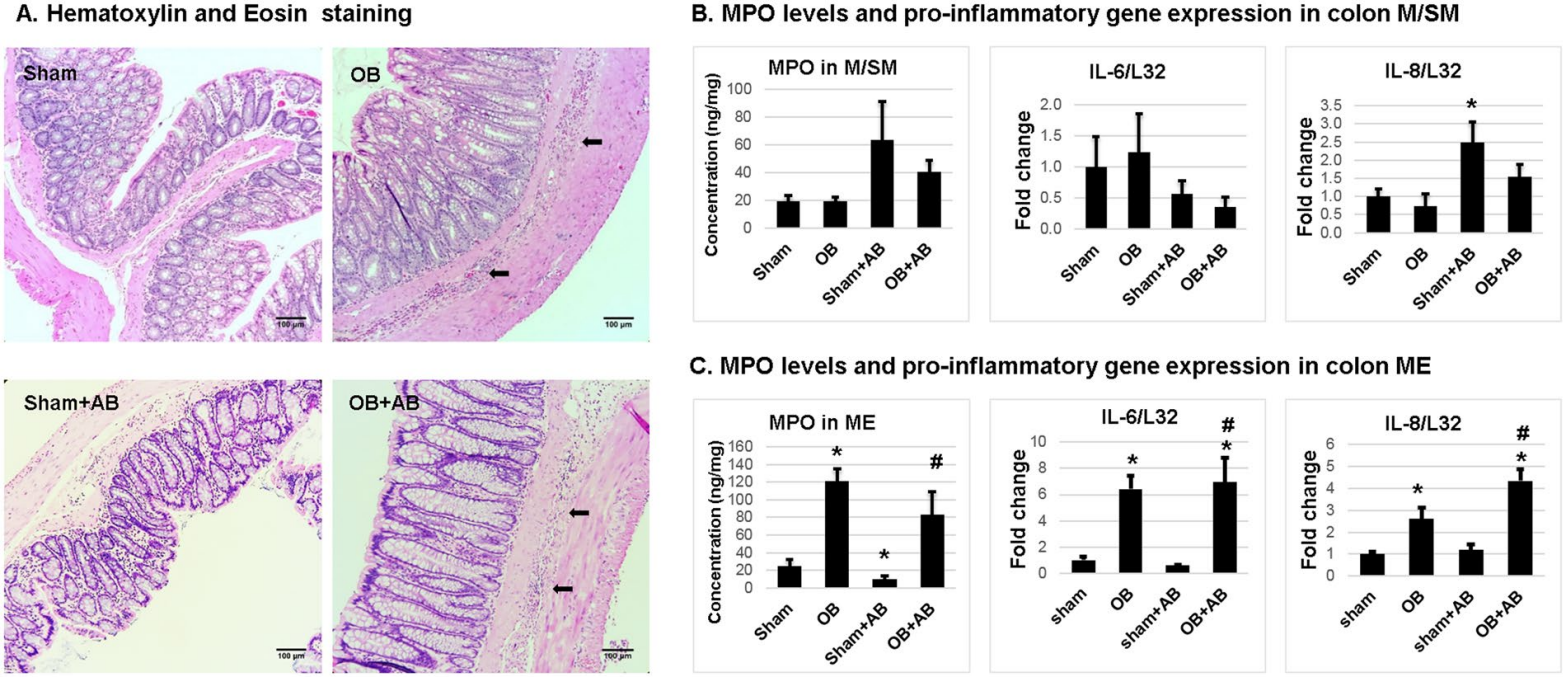
Inflammatory changes in the colon of sham and OB rats without and with antibiotic treatment. Inflammatory infiltration (marked with arrow) in full thickness colon sections of control sham and OB rats and antibiotics treated sham and OB (+AB) rats was assessed by hematoxylin and eosin staining (**A**). MPO levels were quantified using ELISA in colonic mucosa/submucosa (M/SM) (**B**) and muscularis externae (ME) (**C**) of control and antibiotics treated sham and OB rats. Quantitative RT-PCR analysis of IL-6 and IL-8 mRNA expression in colon M/SM (**B**) and ME (**C**) collected from rats treated with and without antibiotics treatment. *p < 0.05 Vs sham, ^#^*p* < 0.05 Vs sham + AB (1-way ANOVA), n = 5 in each group except for sham and OB samples from M/SM, where n = 6.

We determined the inflammatory status in the mucosa/submucosa (M/SM) and muscularis externae (ME) of the mid colon in sham and OB rats with and without antibiotic treatment. OB did not cause significant mucosal inflammation (Fig. 7A,B). Myeloperoxidase (MPO) levels and cytokine expression in colon M/SM were not significantly different between control sham and OB (Fig. 7B). However, in the colonic ME, there was increased inflammatory infiltration in OB animals (Fig. 7A,C), with MPO and mRNA expression of IL-6 and IL-8 reaching significantly elevated levels in the ME in rats with obstruction (*p* < 0.05) (Fig. 7C). Antibiotic treatment did not significantly alter the inflammatory status in the colon M/SM and ME of OB rats (Fig. 7C), although it slightly increased mRNA expression of IL-8 in the M/SM in sham rats (p < 0.05) (Fig. 7B).

### Effects of antibiotic elimination of microbiota on colon smooth muscle contractility in sham and OB rats

**Figure 8:**
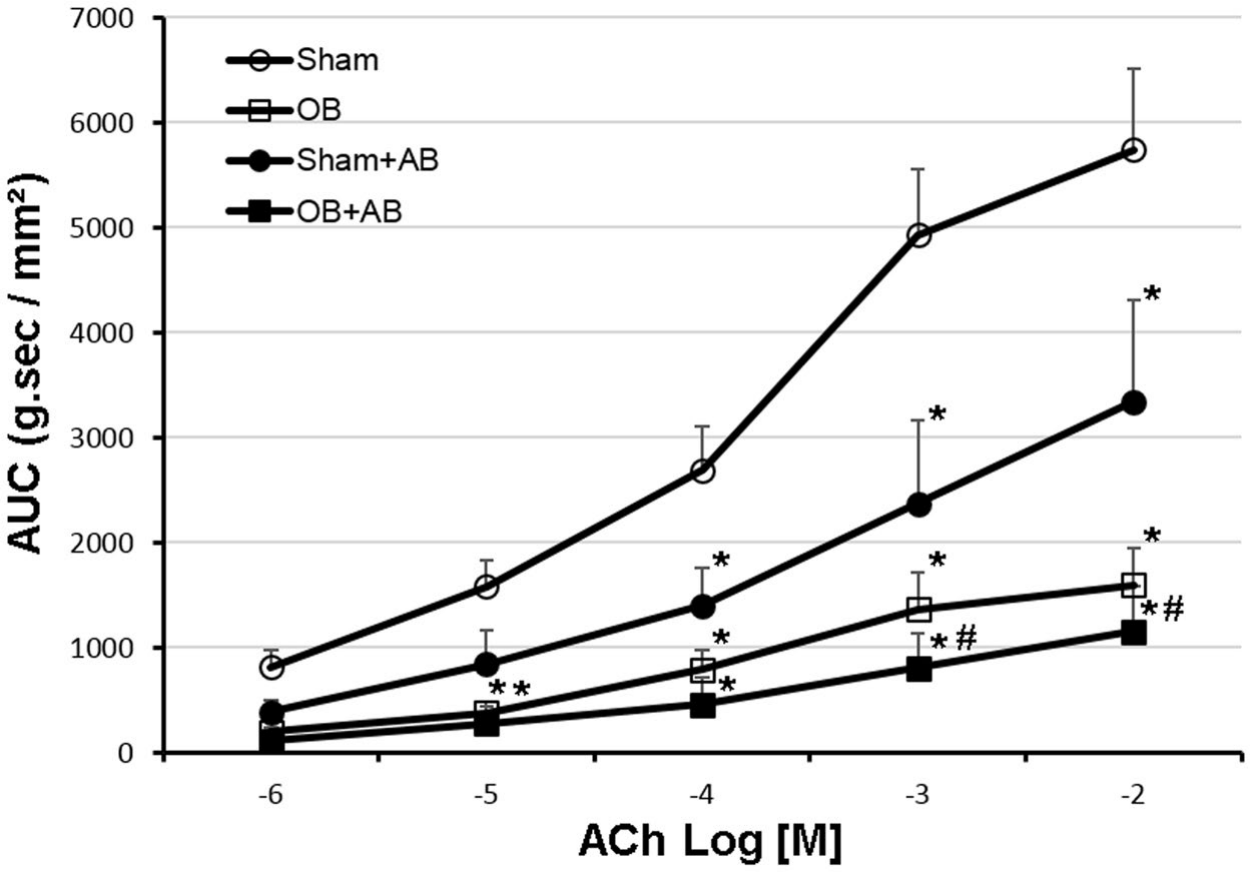
Colon smooth muscle contractility of sham and OB rats without and with antibiotic treatment. Smooth muscle contractility in colon muscle strips dissected from sham (round clear), OB (square clear), antibiotic treated sham (sham + AB, round solid) and antibiotic treated OB rats (OB + AB, square solid) was determined in muscle bath. Quantitative data are represented as area under curves (AUCs) after normalized using the cross section area of each strip. The figure depicts mean AUCs ± standard error. *p < 0.05 Vs Sham, ^#^*p* < 0.05 Vs sham + AB (2-way ANOVA), n = 4 except for sham where n = 5.

To determine whether gut microbiota plays a role in smooth muscle function, we compared the contractile response of colonic circular smooth muscle in sham and OB rats treated with and without antibiotics. Consistent with previous findings^1,29,30^, colonic smooth muscle contractile response was dramatically suppressed in OB. However, antibiotic treatment did not improve colon muscle contractility in OB (Fig. 8). Interestingly, antibiotic treatment resulted in significantly reduced muscle contractility in sham rats (*p* < 0.05), suggesting that microbiota may play an important role in maintaining smooth muscle contractile function in normal conditions (Fig. 8).

## Discussion

Bowel obstruction is a rather common GI disorder, which leads to retention of luminal contents in the segment proximal to the site of occlusion. Bacterial overgrowth has long been thought to occur in the obstructed segment, and to contribute to local gut dysfunctions and systemic responses^9^. This is why broad-spectrum antibiotics are routinely used in the management of bowel obstruction. In the present study, we found no significant increase in the bacterial CFU or total 16S rRNA gene copy number in obstruction. Thus, our results suggest that there is no bacteria proliferation in the obstructed colon. However, we found significant changes of microbiota composition in OB at all the tested taxonomic levels. At the phylum level, the relative abundance of *Firmicutes* is decreased, whereas *Proteobacteria* and *Bacteroidetes* are increased in OB. These changes in microbiota composition at phylum level are consistent with the findings in Hirschsprung’s disease and constipation, resembling chronic functional obstruction in the distal bowel^31,32^. In the murine model of Hirschsprung’s disease, the endothelin receptor B-null (Ednrb−/−) mice have colorectal aganglionosis^31^. Thus, the terminal bowel is constricted as the site of obstruction and the proximal segment becomes distended, resembling chronic OB. Heterozygote mice, alike wild-type mice, have a normal colon and intact enteric nervous system. Studies in the murine model of obstruction found that mutant mice at the age of 20~24 days, when bowel distension was apparent, contained less *Firmicutes* and more *Bacteroidetes* and *Proteobacteria* in the colon feces than wild-type or heterozygote mice. The authors concluded that aganglionosis disrupted the normal colonic microbiome, and that enteric nervous system is an important determinant of microbiome composition. However, we found that the microbiota dysbiosis in the model of mechanical OB, which has an intact enteric nervous system, is similar as in the aganglionosis mice model. Our data suggest that bowel obstruction itself, rather than the absence of enteric neurons, may be the cause of dysbiosis. It is well recognized that multiple environmental and host factors contribute to gut microbiota composition^33,34^. Although sham and OB rats were treated similarly in our study in terms of daily handling and diets, the luminal homeostasis is different in obstruction in many ways i.e. intraluminal pressure, pH, osmolality, mucosa integrity, and gut motility. It is yet to determine what factor(s) may have accounted for the changes of microbiota in the obstructive condition.

Several studies have attempted to correlate microbiota composition changes with gut inflammation and related symptoms. Increases in *Proteobacteria* and *Enterobacteriaceae* have been reported in gut inflammation (i.e. IBD and necrotizing enterocolitis) and functional disorders such as IBS, and are implicated in the pathophysiology of these disorders^35–40^. The altered relative abundance of *Lactobacillus* has also been observed in IBD and IBS^41,42^. Recent studies discovered that *Lactobacillus* levels were decreased in both diarrhea and constipation dominant IBS^16,43^. We detected similar changes of microbiota composition in the model of mechanical obstruction. However, it appears that the alterations of microbiota composition were not associated with inflammatory changes in our model of mechanical OB. In fact, we did not find any obvious inflammation in colonic mucosa in OB, though moderate inflammation was detected in the deeper muscularis externae layer of obstructed colon. Moreover, antibiotic treatment did not significantly change the inflammatory status in the mucosa and muscularis externae layers in the OB rats. The inflammatory changes in the muscularis externae in OB may be due to the effect of mechanical stress on inflammatory gene expression in the gut wall. Previous studies showed that obstruction-associated lumen distention represents a static mechanical stretch to the gut wall and induces up-regulation of inflammatory mediators, cytokines and chemokines specifically in gut smooth muscle cells^10,44,45^. Thus, it is yet to determine what pathological features lead to the changes of microbiota composition, and whether the microbiota composition changes play a pathophysiological role in producing various symptoms in IBD, IBS, and OBD. However, recent studies using humanized microbiota ex-germ free mouse models are providing evidence that dysbiotic microbiota communities indeed have the potential to modulate disease in animal models of IBD, IBS and antibiotic-associated diarrhea^46–48^. Similar studies are now required to assess whether dysbiotic microbiota communities trigger OB-associated dysfunctions in animal models.

Microbiota richness (greater diversity) is thought to be associated with the host’s gut health. A reduction of microbiota diversity was reported in several GI disorders^17,19,21^. However, we found that the microbiota diversity is greater in the obstructed colon than in sham controls. This finding is consistent with the results in the model of chronic functional bowel obstruction^31^ and in functional GI disorder such as constipation^49^. Recent clinical studies found a profound negative correlation between microbiota richness and colon transit rate (assessed by Bristol stool score), with the maximum of species richness in subjects with fast colon transit and the minimum in the slow transit individuals^49^. The increased bacterial species diversity observed in OB may be a result of slower transit rate in the obstructed colon. These studies challenge the current view of high richness being directly associated with gut health of the host.

To assess the possible role of microbiota in local and systemic dysfunctions in OB, we administered antibiotics to eliminate microbiota in sham and OB rats. Increased bacteria translocation in OB is thought to contribute to systemic responses, i.e. sepsis and septic shock^3,5^. We found OB led to marked increases of bacteria translocation to the MLN, spleen, liver, and blood in our model. The majority of translocated bacteria to the MLN were *Enterobacteriaceae*. This is consistent with previous investigations in patients with bacteria translocation through the gut, in which *Enterobacteriaceae* was the most commonly identified bacteria and was found in 55% of the patients with positive culture in MLN^50^. Treatment with broad-spectrum antibiotics eliminated bacteria in the spleen, liver and blood. However, bacterial translocation to the MLN was not affected by antibiotics. Previous reports described MLN as a protective niche of enteric pathogens during antibiotic treatment, as these pathogens residing in the MLN may contribute to relapsing infections^51,52^. Together, our results suggest that broad-spectrum antibiotics have limited effects in blocking bacteria translocation in OB. Characterization of microbiota alterations in OB may help to develop antibiotics and probiotics to specifically target dysbiosis in obstruction.

Gut motility function is severely compromised in bowel obstruction^3–5^. Our previous studies demonstrated that mechanical stretch-induced expression of cyclo-oxygenase-2 (COX-2) in colonic smooth muscle cells plays a critical role in motility dysfunction in OB^1,29^. Lin *et al*. showed that COX-2 derived prostaglandins (PG), i.e. PGE_2_ through EP2 and EP4 receptors, account for suppression of muscle contractility^30^. The present study also found that colonic smooth muscle contractility was significantly suppressed in control OB^1,29,30^. Antibiotic elimination of microbiota did not improve muscle contractility in the OB rats. However, we found that antibiotic elimination of gut microbiota significantly reduced colon smooth muscle contractile response in the sham control animals compared to those without antibiotic treatment. This data is consistent with previous findings that gut motility function is compromised in germ-free animals^53,54^ and by antibiotic eradication of microbiota^55^. However, this may also reflect an adverse response to the antibiotic-induced dysbiosis, which is predominated by resistant pro-inflammatory *Proteobacteria*. Recent studies suggest that gut microbes and their products may act on the enteric nervous system and endocrine cells to influence gut motility^56,57^. However, the precise mechanisms for normal gut microbiota in maintaining smooth muscle function are yet to be examined.

It is noteworthy that although antibiotic treatment drastically decreased the total microbiota population in the colon in both sham and OB rats, it has different effects on different genera of bacteria. Multiple studies have reported that antibiotic treatment may induce overgrowth of pathogenic bacteria in the gut^58,59^. The proposed mechanism is the loss of antibiotic-sensitive beneficial bacteria, which would have helped in maintaining a healthy composition of the gut microbiota. In the absence of these beneficial bacteria, the antibiotic-resistant bacteria can proliferate and disseminate. In support of the theory, we observed a significant decrease in the abundance of *Bacteroidia* which are known to play an active role in maintaining healthy gut homeostasis^60,61^, whereas relative abundances of potentially harmful *Proteobacteria* and *Enterobacteriaceae* were increased after antibiotic treatment. Thus, our data raise concerns about the use of broad-spectrum antibiotics in non-complicated obstruction and call for exploration of obstruction-specific antibiotics and probiotics.

In summary, partial OB led to microbiota dysbiosis in the colon with a decreased relative abundance of *Firmicutes*, especially *Lactobacillus*, and increased abundance of *Proteobacteria* and *Bacteroidetes*. Obstruction also increased colon microbiota diversity. However, OB did not increase total bacterial abundance. Obstruction increased bacterial translocation, suppressed smooth muscle contractility, and led to moderate inflammation in colonic muscularis externae, but not in the mucosa. Antibiotic elimination of gut microbiota increased relative abundance of *Proteobacteria* in the colon, but had limited effects on OB-associated changes in bacterial translocation, muscle contractility, and gut inflammation. However, our study suggests that microbiota may play a role in maintaining normal smooth muscle contractility in the colon.

## Materials and Methods

### Induction of partial colon obstruction in rats

The Institutional Animal Care and Use Committee (IACUC) at the University of Texas Medical Branch (UTMB) approved all procedures performed on the animals. All the experimental methods were performed in accordance with the Guide for the Care and Use of Laboratory Animals of the National Institutes of Health, USA.

Male Sprague-Dawley rats weighing 190–260 g (Harlan Sprague Dawley, Indianapolis, IN) were used in the study. The rats were housed in a controlled environment (temperature, 23 °C ± 2; Humidity 55% ± 10; 12-h light/dark cycle) and allowed free access to food and water throughout the study. Partial colon obstruction was surgically induced as previously described^1,30,62^. Briefly, after rats were anesthetized with 2% isoflurane inhalation, a midline laparotomy incision of about 2 cm in length was made and the distal colon was exposed. A medical-grade silicon band of 3 mm wide and 20 mm long was placed around the distal colon, approximately 2–3 cm from the anus. Obstruction was maintained for 7 days. Control rats also underwent the laparotomy as above, but the band was removed immediately after placing.

### Sample collection, DNA isolation and 16S rRNA gene sequencing of fecal microbiota

Rats were euthanized using CO_2_ inhalation, and fecal contents in the mid colon (~2 cm above the obstruction band) were collected aseptically and immediately snap frozen in liquid nitrogen. Samples were stored at −80 °C until further use. Fecal bacterial DNA was isolated using a MoBio PowerFecal kit (MoBio, USA) according to the manufacturer’s guidelines. The isolated DNA was amplified using universal 16S rRNA V3-V4 region primers^63^. Sequencing was performed with an Illumina MiSeq instrument resulting in 20,000–140,000 base pair paired-end reads according to the manufacturer’s guidelines. The raw sequencing reads were trimmed to 300 bases and filtered to exclude reads with low quality, two or more unknown characters, sequencing adapters. To identify the presence of known bacteria, the subsequences were analyzed using CLC Genomics Workbench 9.5 Microbial Genomics Module. Reference based OTU picking was performed using the SILVA SSU v123 database with 97% sequence identity^64^. Sequences present in more than one copy but not clustered to the database were then placed into de-novo OTUs (97% similarity) and aligned against the database with 80% similarity threshold using MUSCLE (Multiple Sequence Comparison by Log- Expectation) alignment^65^. Alpha diversity was estimated using Shannon diversity index at genus level^66–68^ and statistical significance was calculated using Mann-Whitney test.

Total relative abundance percentage was calculated using mean relative abundance across the samples. Statistical comparison between the groups was performed using two-way ANOVA in GraphPad Prism software (GraphPad Software, Inc. La Jolla, CA, USA) and *p* values were corrected with Sidak’s multiple comparison test.

Linear discriminant analysis (LDA) effect size (LEfSe) was calculated (http://huttenhower.sph.harvard.edu/lefse/) to identify the genera differentially represented in sham and OB rats^28^. LEfSe first performs statistical analysis to calculate significant differences among biological classes and then does additional tests to evaluate whether the observed differences are consistent with expected biological behavior. LEfSe analysis was performed with an α value of 0.05 for the factorial Kruskal-Wallis test and an LDA score effect size threshold of 2 for the first 150 highly abundant genera in Sham and OB.

### Treatments with antibiotic cocktail

In the experiments involving antibiotic (AB) treatment, both sham and OB animals received daily gavages of 1 ml antibiotic cocktail solution throughout the experiment starting from one day before the laparotomy. Antibiotic cocktail consisted of metronidazole, ampicillin, and kanamycin each at 100 mg Kg^−1^ body weight, and vancomycin 50 mg Kg^−1^ as described before^69^.

### Quantitative RT-PCR analysis of colon feces

Total DNA was isolated from pre-weighed colon feces using QIAGEN stool mini kit according to the manufacturer’s instruction for bacterial DNA isolation. Briefly, fecal samples were homogenized in 1.4 ml ASL buffer by vortexing thoroughly and boiled at 95 °C for 5 min to facilitate the lysis of gram positive bacteria. DNA containing supernatants were treated with inhibitEX tablets provided with the kit to eliminate possible PCR inhibitors. After proteinase K treatment, DNA was extracted and quantified.

16S rRNA primers used in the study are as follows: For universal bacteria, UniF: GTGCTGCATGGTCGTCGTCA; UniR: ACGTCGTCCACACCTTCCTC (T_m_: 60 °C, R^2^: 0.994)^70^. Bacterial 16S rRNA regions were amplified by taking 100 ng feces DNA as template, 25 pmol/μl specific primers and 1X power SYBR green master mix (Applied Biosystems, Foster City, CA USA) in 20 μl final reaction volume. All the PCR were done at least in duplicates in StepOne plus Real-time PCR system (Applied Biosystems, Foster City, CA USA). The PCR conditions were as below: 95 °C for 10 min, followed by 40 cycles at 95 °C for 30 s, annealing for 30 s at respective T_m_ temperature, and 72 °C for 45 s. Melting curve analysis was carried out at the end.

The standard curve for universal bacteria quantification was generated by serial dilution of pJet plasmid (Invitrogen, Waltham, MA USA) in which 16S rRNA target sequence from *E.coli (DH10B)* was cloned. Copy number for the standard curve was calculated using the below formula: [A × concentration of DNA (g l^−1^) × volume of DNA in liter]/[molecular weight of plasmid in (g mol^−1^)], where A was the Avogadro constant (6.02 × 10^23^). The total bacterial 16S rRNA gene copy numbers/mg of sample was calculated using the following equation, as reported before^71^. Copy numbers/mg = [Mean Copy numbers × DNA concentration (ng μl^−1^) × Dilution volume of extracted DNA (μl)]/(ng of DNA taken for analysis × mg of sample used for DNA isolation).

### Enumeration of total anaerobic and aerobic bacteria in colon feces

Microbiological enumeration was carried out from pre-weighed fresh feces. The samples were homogenized immediately after collection in Gifu anaerobic media (GAM) (HIMEDIA, West Chester, PA USA) by vortexing and using micro tube homogenizer (Bel-Art, Wayne, NJ USA). Serial dilutions of the samples were plated on GAM agar plates for quantification of anaerobic bacteria. Serial dilutions were also plated on tryptic soy agar (TSA) plates (Difco, BD, Franklin Lakes, NJ USA) for enumeration of aerobic and facultative bacteria. GAM plates were incubated in anaerobic jars containing anaerogen bags (Sigma, St. Louis, MO USA), whereas TSA plates were incubated in 5% CO_2_ at 37 °C. After 24 h, plates were counted for visible bacterial colonies, and colony forming units (CFU)/gram sample taken were calculated.

### Bacterial translocation study

The spleen, liver, and mesenteric lymph nodes (MLN) were collected from rats aseptically using sterile instruments immediately after the euthanasia. The tissues were surface sterilized by short flaming and homogenized in GAM broth. The samples were incubated on ice for 1 h and centrifuged at 1500 rpm for 5 min. Appropriate dilutions of the supernatant were plated on GAM and TSA agar, and plates were incubated as described above. Blood was collected aseptically from ventricular puncture and supplemented with sodium citrate anticoagulant. Red blood cells were separated by centrifuging at 10,000 rpm for 10 min and appropriate dilutions were used for the plating. Bacterial translocation was measured by counting the total aerobic and anaerobic CFU in respective agar plates and represented as total CFU per gram tissue or per ml blood.

### Measurement of colonic circular muscle contractility

Colonic circular smooth muscle strips of 3 × 10 mm were prepared from the mid colon segment ~2 cm oral to the obstruction band^1,62^. Muscle strips were mounted along the circular muscle orientation in muscle baths (Radnoti Glass, Monrovia, CA, USA) containing 10 mL carbonated Krebs solution. The muscle contractility was recorded as described before^1,62^ using Grass isometric force transducers and amplifiers connected to Biopac data-acquisition system (Biopac Systems, Goleta, CA, USA). The equilibration of muscle strips were done at 1 g tension for 60 min at 37 °C, and then contractile response to acetylcholine (ACh) 10^−6^–10^−2^ M was recorded with 15 to 20 min interval. Contractility was measured in terms of the increase in area under curve (AUC) in the first 4 min of ACh addition against the AUC during 4 min before the addition of ACh. AUCs of each muscle strip were normalized with its cross section area which was calculated using below formula: wet tissue weight (mg)/[tissue length (mm) × 1.05 (muscle density in mg/mm³)].

### Colon tissue collection, RNA isolation and quantitative RT-PCR

The mid colon segments were collected in fresh carbogenated Kreb’s buffer. The muscularis externae (ME) was separated from the mucosa/submucosa (M/SM) layer by microdissection as described previously^1,2,30^. Tissues were snap frozen and stored in −80 °C until use. Total RNA was extracted from tissues using Qiagen RNeasy kit (Qiagen, Valencia, CA). cDNA was synthesized from 500 ng of total RNA using the SuperScript III First-Strand Synthesis System (Invitrogen, Carlsbad, CA). Real-time RT-PCR was performed with an Applied Biosystems 7000 real-time PCR system (Foster City, CA) using a SYBR Green PCR master mix (Life Technologies, Grand Island, NY). Relative quantitation of gene expression was done with reference to endogenous control (L32). The primer sequences for the real-time RT-PCR assays are as follows: L32 F-ttgctcacaatctgtcctctaagaa; L32 R-cgttgggattggtgactctga; IL-6 F-caaagccagagtccattcagagc; IL-6 R-ggtccttagccactccttctgt; IL-8 (CINC-1) F- gccacactcaagaatggtcg; IL-8 (CINC-1) R-gacgccatcggtgcaatcta.

### Myeloperoxidase (MPO) assay

Rat colonic ME and M/SM layers were homogenized separately in cold PBS supplemented with protease inhibitors for protein extraction. The MPO content in the protein extract was measured with an MPO EIA kit purchased from HyCult Biotechnology (Udem, The Netherlands) according to the manufacturer’s instruction^1,44^. The assay results were read using accuScan FC system from Fisher-Scientific (Suwanee, GA, USA).

### Histological study

Full-thickness mid colon segments 2 cm oral to obstruction were fixed in 10% buffered formalin for 48 h. Sections of 4-µm thickness were prepared after paraffin embedding, and conventional hematoxylin and eosin-staining was done at the University of Texas Medical Branch Histopathology Core as described before^1,44^. Images were taken using Revolve light microscope from Echo labs (Echo, San Diego, USA).

### Statistical analysis

Graphs were generated using GraphPad Prism (GraphPad Software, Inc. La Jolla, CA, USA) or in MS Excel. Results are represented as means ± standard deviation unless stated. Comparison between the sham and OB samples were done using two tailed unpaired *t*-test assuming unequal variance. Multiple comparisons were done using one way ANOVA and *p* values < 0.05 were considered significant.

## Electronic supplementary material

Supplementary Information

## Data Availability

The datasets generated during and/or analyzed during the current study are available from the corresponding author on reasonable request.
