# Supplementary material for: Microbiota dysbiosis and its pathophysiological significance in bowel obstruction
Source: Sci Rep. 2018 Sep 3;8:13044. doi: 10.1038/s41598-018-31033-0 (PMC6120911; doi:10.1038/s41598-018-31033-0)

Microbiota dysbiosis and its pathophysiological significance in bowel obstruction

Hegde, Shrilakshmi [1]; Lin, You-Min [1]; Golovko, George [2]; Khanipov, Kamil [2]; Cong, Yingzi [3]; Savidge, Tor [4]; Fofanov, Yuriy [2]; and Shi, Xuan-Zheng [1, #]

1. Dept. of Internal Medicine, University of Texas Medical Branch, Galveston, TX, USA.
2. Dept. of Pharmacology & Toxicology, University of Texas Medical Branch, Galveston, TX, USA.
3. Dept. of Microbiology & Immunology, University of Texas Medical Branch, Galveston, TX, USA.
4. Dept. of Pathology & Immunology, Baylor College of Medicine, Houston, TX, USA.

Address for communication:
Dr. Xuan-Zheng Shi, Department of Internal Medicine, Division of Gastroenterology, University of Texas Medical Branch, 4.106 Basic Science Building, 301 University Boulevard, Galveston, TX 77555-0655. Phone: (409) 772-0940. FAX: (409) 747-3084. E-mail: xushi@utmb.edu

Supplementary figure 1 (Fig. S1): 16S rRNA sequencing data showing the mean relative abundance of all the genera identified with significantly different abundance in OB compared to sham (Sham: n=7, OB: n=8; 2-way ANOVA, p<0.05).

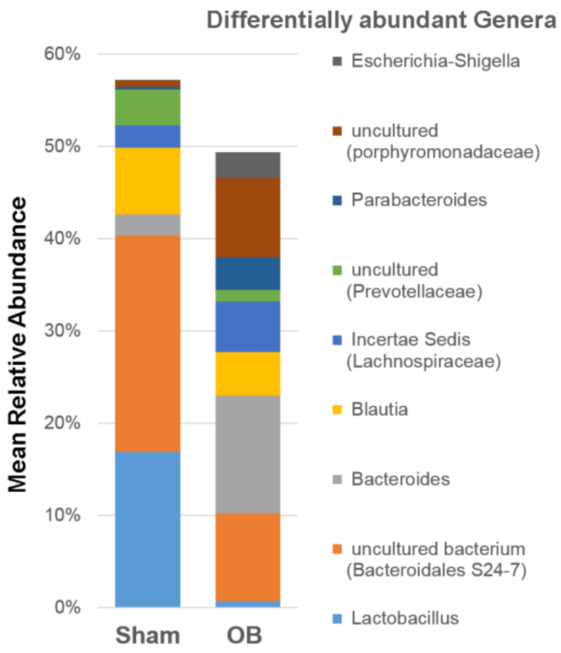

Supplementary figure 2 (Fig. S2): Microbiota alpha diversity at genera level in sham and OB rats with antibiotic treatment (A), compared to the respective counterparts without antibiotic treatment (B and C). Alpha diversity was calculated using the Shannon Diversity Index and statistical significance between the groups was calculated using Mann-Whitney test in Graphpad Prism, and $p<0.05$ is considered significant.

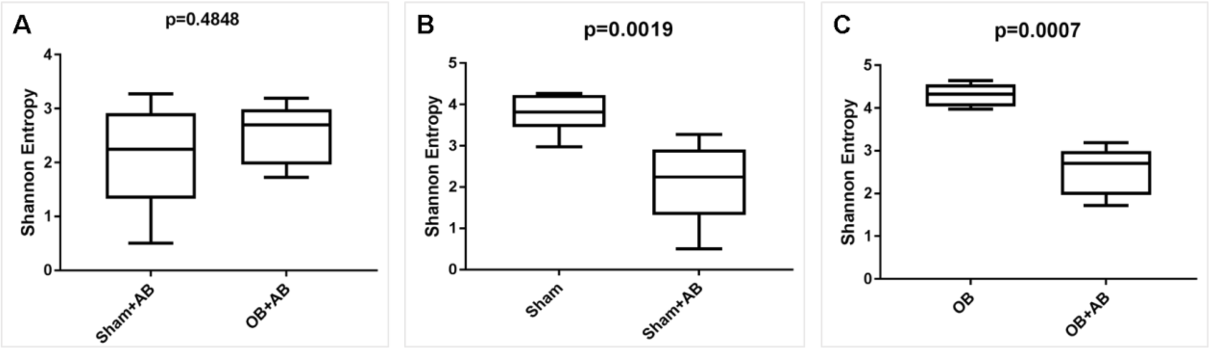

Supplement: Supplementary file 1 — Supplementary Information [file 41598_2018_31033_MOESM1_ESM.pdf]
